# Management of Fracture-Related Infection in Conflict Zones: Lessons Learned from Medical Missions to Gaza

**DOI:** 10.3390/antibiotics13111020

**Published:** 2024-10-30

**Authors:** Elias Nasser, Nour Alshaer, Muaaz Wajahath, Bilal Irfan, Mohammed Tahir, Mosab Nasser, Khaled J. Saleh

**Affiliations:** 1 UT Southwestern Medical Center, Dallas, TX 75390, USA; elias.nasser@utsouthwestern.edu; 2 FAJR Scientific (NGO), Houston, TX 77041, USA; 3 School of Medicine, The Islamic University of Gaza, Gaza P.O. Box 108, Palestine; 4 College of Human Medicine, Michigan State University, East Lansing, MI 48824, USA; 5 Harvard Medical School, Boston, MA 02115, USA; 6 College of Medicine, Central Michigan University, Mount Pleasant, MI 48859, USA; 7 School of Medicine, Wayne State University, Detroit, MI 48202, USA

**Keywords:** fracture-related infection, orthopedic trauma, conflict zones, medical mission

## Abstract

**Background/Objectives**: Fracture-related infections (FRIs) are a significant complication in conflict zones, where limited resources and damaged infrastructure complicate orthopedic care. **Methods**: This study retrospectively reviews the management of FRIs during medical missions to Gaza from April to July 2024. **Results**: Among 135 patients treated for war-related fractures, 30% were identified with suspected FRIs, which were primarily following explosive injuries. Contributing factors to the high incidence of infection included malnutrition, poor sanitation, and the scarcity of sterile surgical supplies. The absence of standard infection control measures further complicated treatment. **Conclusions**: These findings highlight the critical need for a comprehensive approach that incorporates infection prevention, sustainable healthcare planning, and quality assurance tailored to the realities of conflict zones. The study underscores the importance of international support to ensure the availability of essential medical supplies and to develop effective, context-specific strategies for infection management. By applying these insights, healthcare providers can improve patient outcomes and reduce the burden of FRIs in resource-limited settings affected by conflict.

## 1. Introduction

The demand for orthopedic trauma care poses a significant challenge to patients from low- and middle-income countries (LMICs). Orthopedic trauma has been estimated to affect nearly 3 million people worldwide, far exceeding the incidence and mortality of HIV/AIDS and other infectious diseases [1]. Global trauma care is heavily constrained by resource and infrastructure limitations, resulting in surgical environments with inadequate infection-prevention protocols, which further complicate the outcomes of musculoskeletal injuries in underserved populations. The burden of orthopedic trauma care disproportionately affects LMICs and global conflict zones, which has been previously reported in countries like Libya, Yemen, and Gaza [2,3,4].

The ongoing humanitarian crisis in Gaza has introduced unique obstacles to both local and visiting providers. Over the past nine months of war, health infrastructure has become a notable target with junior physicians and medical students forced to lead efforts in providing surgical services for the local population [5,6]. Not-for-profit organizations have since taken the necessary steps to support the decimated health system by delivering medical supplies and medications, sending volunteer surgeons, and providing monetary compensation for overworked local staff who have not been paid since the beginning of the war. Visiting surgeons have cited infection control as a major obstacle for delivering safe surgical care. Poor sanitation, overcrowding, and acute malnutrition have induced a devastating surge of infectious diseases and postoperative infection on the entire population of Gaza with children facing increased incidences of diarrheal diseases, respiratory illness, and hepatitis A [7,8,9,10,11]. Pediatric populations in Gaza suffer high rates of polytrauma and frequently require amputations following infection, which is most often due to the limited supply of antibiotic therapy [12].

Fracture-related infection (FRI) is a serious complication in the management of orthopedic trauma. The risk of FRI following internal fixation is estimated to be less than 2% for closed fractures and steadily rises with extensive soft tissue damage with rates of up to 30% in open fractures [13]. *Staphylococcus aureus* and Coagulase-negative staphylococci are responsible for more than half of all FRI, which are followed by *Enterobacteriaceae* and *C. acnes* [14]. Biofilm formation by the aforementioned microorganisms on foreign material further complicates the management of FRI due to increased risk of resistance to antimicrobial therapy [15]. The diagnosis and treatment of FRI become particularly challenging in resource-constrained settings where laboratory, histopathology, and microbiology testing are often unavailable [16].

The diagnosis and management of FRI involves a series of clinical and laboratory studies. Depypere et al. outlined suggestive and confirmatory signs and symptoms for identifying FRI [17]. Clinical signs and symptoms suggestive of infection include localized redness and swelling, joint effusion, or wound drainage. Increased serum inflammatory markers (ESR, CRP) and/or radiological indication for nonunion can also be used as suggestive criteria for infection. Confirmatory studies for FRI-associated infection include the presence of pus, microbiology culture (from a minimum of two deep tissue samples), and at conventional histological examination, evidence of microorganisms on special stains and/or the presence of more than five PMN/HPF [18]. In 2018, an international consensus on the definition of FRI was established, building on the criteria discussed earlier [19,20]. In conflict zones where confirmatory criteria such as microbiology cultures and histopathology results cannot be collected, providers depend exclusively on suggestive criteria such as evidence of purulent drainage from wounds or frank pus present during surgery. The purpose of this study is to outline the challenges in recognizing FRI in conflict zones and provide recommendations for the management of wound and hardware infection after fracture.

Recent medical missions to Gaza have provided valuable insights into infection control practices and highlighted the severe strain on the healthcare system evidenced by hardships for local medical workers, the overcrowding of hospital corridors, and a critical shortage of sterile supplies [7,21,22]. The escalating violence has overwhelmed hospitals, depleted medical supplies, and increased risks of infections, particularly in patients with fractures [23]. Infection prevention is further complicated by the risk of hematogenous spread, comorbidities, and infections in already injured or multi-fractured patients. Furthermore, the mental health implications of residing in conflict zones underscore the necessity of a comprehensive national strategy to address the well-being of vulnerable populations, especially children and adolescents who may suffer from fractures and other injuries [24]. Orthopedic missions in these challenging conditions highlight the need for quality assurance practices, sustainable planning, and the use of measurable indicators to ensure that the mission’s goals—treating acute injuries and maintaining infection control standards—are effectively achieved [25]. The purpose of this article is to present the orthopedic injury patterns observed in Gaza and describe the current challenges associated with operating in an ongoing war zone. We anticipate that FRI will be a common complication of orthopedic trauma given the widespread malnutrition, limited antibiotic therapies, and poor sanitary conditions. The recommendations offered in this article may benefit visiting surgeons operating without adequate infrastructure and limited supply inventory.

## 2. Results

From April to July 2024, 135 patients were treated with war-related orthopedic trauma, specifically fractures with or without major complications requiring surgery. Most patients were male (84%) with a mean age of 31 years (range of 3 to 75 years). Age could not be attained from 13 (9.6%) patients who were treated acutely during mass-casualty incidents, resulting in the incomplete reporting of some demographic variables. During such incidents, patients often presented to the emergency room unaccompanied by individuals who could provide the aforementioned information. We also note a large gap between the number of male (84%) and female (16.3%) patients who were treated with acute fractures. This discrepancy can be explained by the social practice of sheltering vulnerable populations such as women and children in relatively safe areas such as hospitals and schools, exposing men to a greater risk of injury in combat zones. Men were also more likely to be essential workers such as medics or firefighters operating in settings of escalated military conflict. FAJR volunteers also noted that women in Palestinian culture were less likely to pursue surgical interventions for their injuries. Table 1 includes a comprehensive breakdown of the demographic characteristics of patients included in the study.

Among patients with war-related fractures, 41 (30%) presented with suspected FRI with orthopedic hardware based on radiographic evidence of nonunion or clinical signs suggestive of infection. More than half of the total infections (51%) occurred following explosive injury, though it is likely that more patients with unspecified injuries also suffered trauma due to explosive or shrapnel injury. Mechanism of injury could not be reported in 46 (34%) patients due to staffing shortages during emergency, mass-casualty incidents, limiting the data available to volunteers on the ground. Rapid turnaround and overcrowding within hospital areas during periods of escalation prevent local medical staff from ensuring adequate follow-up, including the reporting of missing data. The mechanism and anatomical distribution of the fractures are presented in Table 2.

**Figure 1:**
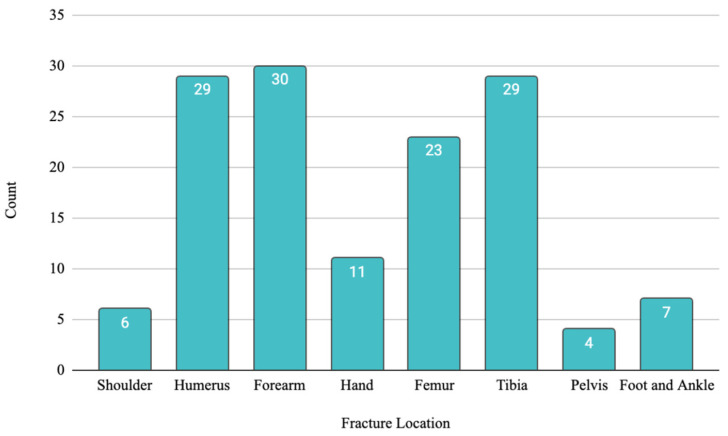
Fracture injury anatomical distribution.

**Figure 2:**
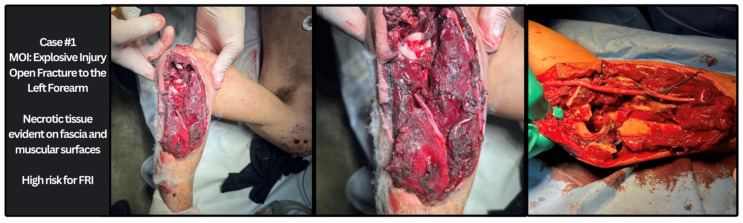
Explosive injury in patient resulting in high FRI risk.

Fractures were also subcategorized by anatomical region (Figure 1) with most fractures occurring in the forearm (n = 30; 22%), humerus (n = 29; 21%), or the tibia (n = 29, 21%). Overall, 22 (54%) patients with FRI were identified with radiological findings of malunion and 17 (42%) presented with purulent drainage or frank pus during exploration surgery; 2 (5%) patients presented with both radiological evidence of malunion and clinically suggestive findings of FRI (Table 3). Open fractures were reported as presenting injury in 21 (16%) patients. Among the fracture injuries identified above, 24 (18%) patients were found to have multiple bone involvement. Some patients demonstrated evidence of both radiological and clinically suggestive signs of infection. Figure 2 offers a sample case from Gaza demonstrating an open fracture of the forearm caused by an explosive injury. According to the classifications described in this article, the patient is suspected to have a high risk of developing FRI without the advised medical therapies, specifically serial irrigation and debridement with antibiotics.

## 3. Discussion

### 3.1. Mechanisms of FRI in Conflict Zones

In this study, more than half of all fractures were caused by explosive injuries. Large explosives are engineered to inflict significant harm on victims by generating a negative pressure suction. These blasts have devastating consequences on civilians both directly and indirectly. The pressure from a blast wave is often lethal with some explosives reaching forces of up to 100 psi. Furthermore, blast waves propagate through surrounding infrastructure, propelling shrapnel and debris to hurricane-like velocities [26]. Haque et al. found that civilians injured by an explosive were more likely to have concomitant injuries and had worse rates of wound closure when compared to patients with gunshot injuries [27]. Moreover, explosive injuries expose patients to penetrating trauma with metals, leaving bone and soft tissue vulnerable to foreign pathogens such as bacteria or fungi [28]. Given the heightened risk of microbial exposure, explosive injuries require careful attention to serial debridement and regular monitoring for early signs and symptoms of infection [29].

Patel et al. reported that the microbiological profile of FRI anatomically favors the tibia (56.9%) and the femur (15.3%) with *S. aureus* as the leading pathogen responsible for infection [30]. Our investigation yielded similar results with the tibia (24%) and femur (21%) making up a significant portion of FRI cases. In contrast, we also found a large number of FRI localized to the forearm (24%) and the humerus (19%). However, due to resource constraints in Gaza, we were unable to classify the microbiological profile of each injury. 

### 3.2. Risk Factors for Developing FRI in Conflict Zones

Treatment failure is an important concern following the diagnosis of FRI. Horton et al. found that evidence of polymicrobial infection, implant removal, and open fractures were the greatest predictors for treatment failure [31]. Many patients in Gaza face long delays in treatment after initial injury, further compounding the risk of treatment failure. Several factors contributed to the delay in treatment for patients with orthopedic injuries. First, there are very few functioning medical centers remaining in Gaza. Second, due to fuel shortages and the destruction of regular roadways, patients often have to travel long distances by foot to reach a hospital [32,33]. This is especially challenging for patients with orthopedic injuries who require continuous physical support. Furthermore, patients risk their safety by traveling through ongoing battlegrounds while fleeing their homes to seek medical care. These adverse conditions induce additional stress upon injured patients, compounding the risk of developing FRI.

Malnutrition and frailty are proven risk factors for complications following orthopedic trauma [34,35,36,37,38,39,40]. While we cannot directly quantify the nutrition levels of patients in Gaza due to limited laboratory infrastructure, we can assume that nearly all Palestinians have experienced some form of structural food insecurity resulting in malnutrition. Since the early stages of war in 2023, the Gaza Strip has been subjected to severe rates of food insecurity at a crisis level (IPC Phase 3) [41]. More than 95% of Gaza’s water has been deemed unsafe for human consumption, and recent reports have cited contamination of the drinking water with poliovirus [10,42]. Access to adequate nutrition and clean drinking water are important for infection prevention and bone healing. Ernst et al. identified malnutrition as a risk factor for postoperative complications and delayed wound healing [36]. Risk of malnutrition has also been shown to contribute to greater rates of morbidity and mortality [40]. Thus, nutritional supplementation and hydration therapy must be prioritized in resource-limited conflict zones.

Upon diagnosis of FRI, systemic deterioration, poor fracture and soft tissue healing, and sepsis are indications for urgent surgical intervention [43]. Metsemakers et al. advise physicians to manage FRI by surgically stabilizing the fracture, treating the infection, and healing the surrounding soft tissue [19]. There are a few treatment strategies for FRI that have been implemented by the Fracture-Related Infection Consensus Group. Immediately following debridement of the infection site, patients should receive no more than 1 week of broad-spectrum intravenous antibiotic therapy followed by oral antibiotic therapy specific to the suspected pathogen. Evidence of purulent drainage or other confirmatory signs of FRI should prompt the immediate initiation of empiric IV antibiotic therapy [44]. The use of intrawound vancomycin or cefazolin powder at the surgical site may also reduce contamination, though surgeons should be concerned with the risk of antibiotic resistance [45,46].

### 3.3. Recommended Practices for Preventing and/or Treating FRI in Conflict Zones

While these treatment modalities have proven effective in standard clinical environments, providers operating in active war zones often lack access to the previously mentioned therapies. Therefore, we have developed a list of recommendations for local and visiting orthopedic surgeons operating in conflict zones [43,44,46,47,48,49,50]. A list of recommendations is presented in Table 4.

### 3.4. Study Limitations

There are several limitations which should be discussed in this study. As a consequence of the war, a reliable electronic medical record was unavailable due to limited internet access and inadequate staff available to monitor patients. Therefore, the data presented in this study do not factor in patient medical histories and surgical outcomes. Additionally, the data provided do not comprehensively include mechanisms of injury or classifications of fracture (closed, comminuted, etc.). Many patients sought emergency care and could not report details regarding their injuries, leading to 46 (34%) patients with unspecified injuries. The data also do not reflect the volume of injuries presenting to Gaza hospitals. Therefore, the data presented in this article may not be extrapolated to represent the patterns of all acute injuries in Gaza. Despite these challenges, the data offer insight into the nature of orthopedic trauma and infection prevalence with a subset of recommendations which may be used to improve the management of FRI in conflict zones.

## 4. Methods

### 4.1. Study Design

This study was developed as a retrospective review of the incidence of orthopedic trauma, specifically fracture, reported during a medical mission trip in the Gaza Strip.

### 4.2. Study Setting

Data were collected during emergency medical missions carried out between April and July 2024 from two large medical complexes located in Gaza: the European Gaza Hospital in Khan Younis and Al-Aqsa Hospital in Deir al-Balah. Overall, 756 patient contacts were made by a single orthopedic and peripheral nerve surgeon volunteering with FAJR Scientific (“FAJR”), a US-based federally designated nonprofit. Local volunteers assisted the surgeon in documenting injuries as they were presented in the emergency room. During this time, local volunteers requested relevant patient demographics such as age and sex. Demographic data such as age and sex were recorded by local volunteers, while the treating physician documented diagnoses and mechanisms of injury prior to surgery. Due to unreliable electricity and limited internet access, local staff were unable to maintain electronic medical records. Instead, data were collected via handwritten notes, which were later transcribed into an electronic medical record system established by FAJR for the mission.

### 4.3. Data Analysis

We retrospectively screened 756 patients for evidence of fractures. The first author classified all patients with confirmed fractures to identify those with fracture-related infections, such as wound or hardware infections. The anatomical distribution of injuries was extracted from the transcribed medical reports using descriptive diagnoses. To minimize bias, a second author independently reviewed and confirmed the data. Parameters analyzed included patient demographics, injury mechanism, clinical assessment, diagnosis, infection status, date of injury, and treatment plan. The study included all patients who underwent surgical treatment for orthopedic war-related injuries. Clinical notes were used to identify patients with orthopedic hardware and those suspected of having infections based on predefined criteria. Patients who opted out of surgery after consultation were excluded as were injuries occurring before 7 October. After applying exclusion criteria, the final sample consisted of 135 patients with acute fractures who received surgical care. The first author identified all cases of radiographic or clinically suggestive fracture-related infections (FRIs) based on diagnoses reported by the treating physician.

### 4.4. Ethics

The study was approved by both the Palestinian Ministry of Health and the FAJR Scientific Ethics Committee. All patient data were deidentified, and no private health information was disclosed.

## 5. Conclusions

The evidence of FRI in Gaza supports the need for intensive infection control efforts and increased availability to antimicrobial therapies. Since the onset of the current escalation, restrictions imposed on Gaza’s borders have severely limited the supply of essential medications, personal protective equipment, and orthopedic tools and implants [51]. It is imperative that international humanitarian efforts focus on the provision of essential medical supplies, including antibiotics and surgical equipment, to mitigate the risk of infection and improve the quality of care delivered to trauma patients in such challenging environments. Visiting medical teams and NGOs working in low- and middle-income countries (LMICs) must also prioritize fostering the autonomy and self-reliance of local staff. Surgeons should focus on training junior staff to ensure the sustainability of local health systems after they leave. Additionally, visiting organizations must be mindful of not straining local resources and should aim to supply surgical materials whenever possible. While each conflict zone offers its own challenges, NGOs have a responsibility to support, accompany, and empower the local medical staff during short-term missions. 

The lessons learned from these medical missions emphasize the critical role of sustainable, long-term strategies in addressing the unique challenges of delivering orthopedic care in conflict zones. By implementing evidence-based practices tailored to the realities of war-torn regions, healthcare providers can better manage FRIs and other trauma-related complications. Future initiatives should prioritize the development of robust, adaptable infection prevention protocols and the establishment of specialized care facilities under a geopolitical environment conducive to building civilian infrastructure and access to medical care in a secure manner, to ensure continuity of care and follow-up for affected populations. These efforts will be crucial in enhancing the resilience of healthcare systems in conflict zones and ultimately improving patient survival and recovery.

## Figures and Tables

**Table 1:** Patients’ demographic data.

Demographic Data	N	%
Age		
Less than 20	24	17.8
20 to 40	70	51.9
Greater than 40	28	20.7
Unspecified	13	9.6
Sex		
Male	113	83.7
Female	22	16.3

**Table 2:** Fracture injury mechanism and anatomical distribution.

Injury Classification	N	%
Mechanism of Injury		
Explosive Injury	68	50.4
Gunshot	18	13.3
Road-Traffic Accident	3	2.2
Unspecified	46	34.1
Anatomical Distribution		
Shoulder	6	4.3
Humerus	29	20.9
Forearm	30	21.6
Hand	11	7.9
Femur	23	16.5
Tibia	29	20.9
Pelvis	4	2.9
Foot and Ankle	7	5.0

**Table 3:** FRI diagnostic criteria and anatomical distribution.

Fracture Related Infection	N	%
Fracture Classification		
Open Fracture	21	15.6
Multiple Bone Involvement	24	17.8
Patients with Suspected FRI	41	29.9
Infection Patients		
Malunion/Nonunion	22	53.6
Clinically Suggestive	17	41.5
Both	2	4.9
Anatomical Distribution		
Shoulder	1	2.4
Humerus	8	19.0
Forearm	10	23.8
Hand	3	7.1
Femur	10	23.8
Tibia	9	21.4
Pelvis	0	0.0
Foot and Ankle	1	2.4

**Table 4:** Recommendations for managing fracture-related infection in resource-limited conflict zones.

No.	Recommendation
1	Administration of prophylactic perioperative and intraoperative intravenous antibiotics such as Cefazolin
2	The use of gentamicin- or vancomycin- loaded bone cements in orthopedic surgeries which the Gaza healthcare system completely ran out of. Doctors on missions have tried to provide some with them, but with the restrictions on the entry of international emergency medical team members and medical aid, they are no longer available there
3	The application of intrawound vancomycin powder with or without stem cell therapy. Hospitals in Gaza have run out of these options
4	Repeat debridement and coverage of soft tissue, and regular change of dressing for infected wounds, and the drainage of pus collection (abscess) either under a local or general anesthetic, along with taking a wound culture swab for pus culture and sensitivity test. Gaza hospitals have been running out of basic components such as petri dishes, flasks, antibiotic solutions and others
5	Use of postoperative prophylactic antibiotics either intravenously or orally such as Amoxicillin with Clavulanic Acid. Medical workers in Gaza have been prescribing these antibiotics as available for all patients postoperatively due to the low sterility of instruments and the field utilized for deep wounds closure
6	Availability of common and broad-spectrum antibiotics which have been sacred in Gaza
7	Use of disposable, sterile, surgical gowns and drapes which are currently completely non-existent in Gaza. Staff at Nasser Medical Complex, largest semi-functioning hospital in Gaza currently, located in Khan Younis, are forced to re-sterilize disposable gowns for surgeries
8	Single use of pins, screws, plates, orthopedic implants and external fixation devices although doctors in the strip have been forced to re-sterilize orthopedic implants and Ex-Fixes for patients which has increased their chances of catching an infection
9	Routine cleaning of the operating theater through damp dusting, and following disinfection and sterilization protocols especially for infected cases. It has been challenging to apply these standard protocols in Gaza because of the limited numbers of operating rooms relative to the number of patients in need of surgeries in addition to the shortage of disinfectants. This has led to the appearance of flies inside the operating rooms at close proximity to patients
10	Double gloving of scrubbed surgeons and nurses which has been challenging in Gaza with the limited availability of sterile gloves
11	Conduct regular research for the effectiveness of used available antibiotics and expected patterns of types of bacteria conducting a series of culture data which has been difficult to access due to the lack of a centralized electronic database in Gaza. This emphasizes the importance of the data collected and used for this research amidst the paucity of such data from hospitals in the Strip
12	Set up specialized wound care clinics at a close distance to populations for follow-up care and change of dressings procedures for prevention of infections, prompt responsiveness to wound infections, and alertness for early signs of osteomyelitis
13	Provide clean living environments which have been completely strenuous with the poor waste management, untreated sewage in the grounds and in hospitals, and low availability of cleaning and hygiene supplies. Patients in Gaza have started growing live maggots in their wounds as a result of this. People in Gaza have been more prone to rodent/animal bites and scrapes scratches due to displacing populations to Al Mawasi- sandy or coastal areas
14	Improve immune systems of patients through providing consistent nutritious meals, clean water and stress management. These have all been difficult to achieve in the setting of a warzone with the limitation of availability of food items and safe drinking water which furthers the chances of post-surgical complications and risks of infections

## Data Availability

The data presented in this study are available upon request from the corresponding author. Access to the data is restricted due to privacy concerns and regulations set forth by the Ministry of Health in Gaza during the ongoing war.
